# Detection of Transgenic and Endogenous Plant DNA Fragments and Proteins in the Digesta, Blood, Tissues, and Eggs of Laying Hens Fed with Phytase Transgenic Corn

**DOI:** 10.1371/journal.pone.0061138

**Published:** 2013-04-08

**Authors:** Qiugang Ma, Chunqi Gao, Jianyun Zhang, Lihong Zhao, Wenbo Hao, Cheng Ji

**Affiliations:** State Key Laboratory of Animal Nutrition, College of Animal Science and Technology, China Agricultural University, Beijing, PR China; Wageningen UR Livestock Research, The Netherlands

## Abstract

The trials were conducted to assess the effects of long-term feeding with phytase transgenic corn (**PTC**) to hens on laying performance and egg quality, and investigate the fate of transgenic DNA and protein in digesta, blood, tissues, and eggs. Fifty-week old laying hens (n = 144) were fed with a diet containing 62.4% PTC or non-transgenic isogenic control corn (**CC**) for 16 weeks. We observed that feeding PTC to laying hens had no adverse effect on laying performance or egg quality (*P*>0.05) except on yolk color (*P*<0.05). Transgenic *phy*A2 gene and protein were rapidly degraded in the digestive tract and were not detected in blood, heart, liver, spleen, kidney, breast muscle, and eggs of laying hens fed with diet containing PTC. It was concluded that performance of hens fed diets containing PTC, as measured by egg production and egg quality, was similar to that of hens fed diets formulated with CC. There was no evidence of *phy*A2 gene or protein translocation to the blood, tissues, and eggs of laying hens.

## Introduction

Phosphorus (**P**) is one of the essential mineral elements for all living organisms. However, there is fairly low content of available P in plant-sourced feed ingredients. About 80% of P in cereal plants, including corn is stored as phytate-P [1]. Phytate bound P is poorly utilized by monogastric animals, due to low levels of phytase activity in their digestive tracts [2]. As a consequence, inorganic P is added to diet to meet the P requirement by animals, which leads to the excretion of non-utilizable portion of dietary P and consequent environmental pollution [3]. Exogenous microbial phytase supplementation in low-P diets has been considered to be one of the most effective ways to reduce P output [4]. However, this approach is associated with high production costs for microbial phytase and requirement of special care in feed processing, which limits its extensive commercial use in diet [5].

The cultivation area for genetically modified (**GM**) plants has increased 94-fold under the demands for more efficient agriculture since 1996, reaching 160 million hectares worldwide in 2011 [6]. In 2011, 88% of the corn, 90% of the cotton, and 94% of the soybeans planted in the United States were GM [7]. Advances in agricultural biotechnology have enabled the production of transgenic plants expressing microbial-derived phytase in tobacco seeds [8], soybean [9], and canola [10]. Recently, an *Aspergillus niger* derived phytase expressed in the endosperm of corn was developed by the Chinese Academy of Agricultural Science [11]. As an innovative way of delivering microbial phytase to non-ruminants for better utilization of phytate-bound P, phytase transgenic corn (**PTC**) will allow animal feed producers to eliminate the purchasing for phytase and corn separately, and eliminate the need for mixing the two ingredients together. This saves time, machinery, and labor for animal feed producers. The PTC has been demonstrated by our lab to improve P utilization in poultry fed P-deficient diets [12].

Compared with conventional corn, corn expressing a microbial-derived gene may have more potential for future development [11]. However, since the increasing feeding with GM plants to meat, milk and egg producing animals, safety concerns have been raised with respect to potential adverse effects on animals and humans health [13]–[18]. Consumer-concerned GM plants may pose a perceived risk to health, development of toxicity or the transfer of the recombinant DNA and proteins from diet to livestock and livestock derived products that are consumed by humans.

To our knowledge, no research has been conducted to evaluate the safety of PTC in animals. Therefore, the objectives of this study were to: 1) explore the effects of 16 weeks of long-term feeding with PTC compared with an isogenic parent line corn on laying performance and egg quality, and 2) assess the fate of recombinant DNA and protein in the gastrointestinal tract (**GIT**) and verify the possible transfer to blood, tissues, and eggs of laying hens after consumption of GM corn.

## Materials and Methods

### 1. Ethics Statement

The protocol reviewed and approved by the Animal Care and Use Committee of China Agricultural University. All procedures were carried out in strict accordance with the recommendations in the Guide for Guidelines for Experimental Animals of the Ministry of Science and Technology (Beijing, China), and all efforts were made to minimize suffering.

### 2. Corn and diets

One non-transgenic isogenic control corn (**CC**) and PTC (Beijing, China) were grown, harvested, and stored under the same environmental conditions. To avoid any possibility of contamination, corns harvested from CC and PTC were strictly separated. Nutrient and energy content of both corn varieties have been reported by our previous study [12].

To prevent contamination during diet preparation, the control diet was mixed before the transgenic diet, both as single batches. The instruments were cleaned using dedicated equipment before, between, and after batches. Each diet was assessed by Polymerase Chain Reaction (**PCR**) to confirm the absence (in control) or presence (in transgenic diet) of the *phy*A2 transgenic gene.

All diets were analyzed for nutrients levels (Table 1) as previously described by Gao *et al.* [12]. Phytase activity in diet was determined according to the method reported by Engelen *et al.* [19]. One phytase activity unit (FTU) is defined as the quantity of enzyme that releases 1 µmol of inorganic P per min from 1.5 mM sodium phytate at pH 5.5 at 37°C. The two treatment diets were formulated to contain adequate concentrations of all nutrients required for laying hens according to the National Research Council [20].

**Table 1:** Composition of the diets (as-fed basis).

Item (% unless note)	CC^1^	PTC^1^
Ingredient		
Corn(non-GM)	62.40	—
Corn (GM)	—	62.40
Soybean (non-GM)	25.0	25.00
Corn oil (non-GM)	1.20	0.70
Limestone	8.30	8.55
Dicalcium phosphate	1.50	1.00
Salt	0.30	0.30
DL-Methionine	0.20	0.20
Choline chloride	0.10	0.10
Vitamin and trace mineral premix^2^	1.00	1.00
Zeolete powder	0.00	0.75
Total	100.00	100.00
Nutrient level		
AME (MJ/kg)^3^	11.17	11.17
CP^4^	16.60	16.60
Lysine^4^	0.79	0.79
Methinoine^4^	0.37	0.37
Methinoine+cystine^3^	0.66	0.66
Ca^4^	3.50	3.50
Total P^4^	0.57	0.49
Nonphytate P^4^	0.32	0.28
Phytase activity^4^	28	5019

1 CC = control corn; PTC = phytase transgenic corn.

2 Provided per kilogram of diet: vitamin A, 12,200 IU; vitamin D3, 4,125 IU; vitamin E, 30 IU; vitamin K, 4.5 mg; thiamine, 1 mg; riboflavin, 8.5 mg; calcium pantothenate, 50 mg; niacin, 32.5 mg; pyridoxine, 8 mg; biotin, 2 mg; folic acid, 5 mg; vitamin B12, 5 mg; manganese, 80 mg; iodine, 1 mg; iron, 60 mg; copper, 8 mg; zinc, 80 mg; selenium, 0.3 mg.

3 Calculated values.

4 Determined values.

### 3. Animal housing and management

One hundred and forty-four 50-week-old healthy Hy-Line Brown laying hens (BW = 1.80±0.10 kg), the mean egg production of 85.15±3.29%, were randomly allocated to two dietary treatments. Each treatment had 8 replicates, and each replicate had 3 stainless steel suspended cages (Yanbei Group, Beijing, China) with 3 hens per cage, all fed from a single feeder per cage. The cage size was 45 cm×45 cm×45 cm. Two experimental diets contained corn (62.4%) either from CC or PTC. Prior to the feeding trial, all the birds were fed the CC diet (Table 1) for 2 weeks. After this adaptation period, the birds were fed the CC or PTC diet, respectively, for 16 weeks. The hens were housed in a completely enclosed, mechanically ventilated building, where they were maintained on a 16-h light schedule and allowed *ad libitum* access to experimental diets and water. Room temperature was maintained at 25±2°C.

### 4. Performance and egg quality measurement

From week 1 to week 16, egg weight and egg production were recorded in replicates at 2:30 p.m. every day. Feed intake was recorded weekly, and feed conversion was calculated. Mortality was recorded daily. On week 16, five eggs from each replicate were collected and the yolk color, albumen height, and Haugh units were measured with the Egg Multi Tester EMT-5200 (Robotmation Co. Ltd., Tokyo, Japan). Eggs were tested on EggShell Thickness Gauge (Robotmation Co. Ltd., Tokyo, Japan) to determine eggshell thickness. Eggshell strength was measured with EggShell Force Gauge (Robotmation Co. Ltd., Tokyo, Japan). The egg shape index was measured with the FHK egg shape determinator (Fujihira Industry Co., Tokyo, Japan).

### 5. Blood, intestinal, organ and eggs sampling

On week 16, one laying hen from each replicate with similar live weight was selected for blood collection. Five milliliters of blood was obtained via wing vein into a sterile tube with EDTA (for DNA extraction).

After weighting, one bird from each replicate was sacrificed to allow bleeding and was then hung upside down for 3 min. The last meal was administered 2 h prior to sacrifice. To avoid accidental cross-contamination, the birds fed CC were sacrificed first, followed by birds fed PTC. All the surgical instruments were disinfected with 75% ethanol solution between animal. New disposable gloves were used for each sample. Immediately following sacrifice, the tissues of heart, liver, spleen, kidneys, and breast muscle were excised, trimmed of any superficial fat or blood clots and collected.

Samples of gut contents were collected in the following order: rectal, cecal, ileum, jejunum, duodenal, gizzard, and crop. This order was followed because the target genes and proteins to be detected were assumed to be more concentrated toward the oral cavity because of the degradation activity of the digestive process [21]. In this way, accidental cross-contamination among different guts was avoided. The samples were collected in separate sterile tubes, placed on ice, and stored at −80°C until analysis.

At the end of the study, five eggs were randomly collected from each replicate. All the eggs were stored at 4°C for DNA and protein extraction.

### 6. DNA extraction

Extreme caution was taken during DNA extraction and PCR to avoid cross-contamination. The samples derived from birds fed with CC or PTC was processed in two different places. Pestles, mortars, homogenizer, and place of work were carefully cleaned before every new sample. New disposable gloves were used for each sample.

#### 6.1 Corn and diets

The CC, PTC and diets were prepared by finely grinding in liquid nitrogen using clean, sterile pestles and mortars. DNA from the ground material was extracted using a DNeasy plant Mini Kit (Qiagen GmbH, Hilden, Germany) according to the manufacturer's protocols.

#### 6.2 Intestinal contents

The samples of the undigested contents of the rectal, cecal, ileum, jejunum, duodenal, gizzard and crop were prepared by finely grinding in liquid nitrogen using clean, sterile pestles and mortars. Genomic DNA from intestinal contents was isolated with the QIAamp DNA Stool Mini Kit (Qiagen GmbH, Hilden, Germany) according to the manufacturer's protocols.

#### 6.3 Blood and tissues

All tissues and blood were defrosted, and then parts of tissues were taken by cutting out an internal portion of the tissue with a clean scalpel blade. DNA from bird tissues and blood was extracted using the DNeasy Blood & Tissue Kit (Qiagen GmbH, Hilden, Germany) according to the manufacturer's protocols, respectively.

#### 6.4 Eggs

Eggs were disinfected with 75% ethanol before being cracked. The content of the five eggs from each replicate was homogenized at 10,000 rpm for 1 min using a disperser (Ultra-Turrax model T25, IKA Werke, Staufen, Germany) on ice. Genomic DNA from eggs was extracted according to the method reported by Herman [22] using the DNeasy Blood & Tissue Kit (Qiagen GmbH, Hilden, Germany).

### 7. PCR analysis

In the PCR studies, a corn specific invertase gene *ivr* (226 bp) was used as the positive control to identify maize DNA [23], and a foreign gene *phy*A2 (678 bp) inserted into the PTC was used to monitor transgenic DNA [11]. In addition, the DNA extracts from digesta, blood, tissues, and eggs were analyzed by PCR for a 396 bp region of the chicken ovalbumin (*ov*) gene to ensure the quality and suitability of the extracted DNA for PCR analysis [24]. Three pairs targeting *ivr*, *phy*A2 and *ov* genes were obtained from Invitrogen (Beijing, China), respectively. The primers and PCR conditions used for the detection of *ivr*, *phy*A2 and *ov* genes are outlined in Table 2.

**Table 2:** Primers and PCR conditions used for the detection of target genes in laying hens digesta, blood, tissues, and eggs samples.

Primer name	Sequence (5′-3′)	Specificity	Target gene	Amplicon size (bp)	PCR conditions^1^	Reference
*ivr*-F	CCGCTGTATCACAAGGGCTGGTACC	Plant (endogenous)	invertase gene	226	95°C×5 min	Hurst, *et al.*, 1999
					94°C×30 s	
					61.5°C×45 s	
					72°C×45 s	
					72°C×10 min	
*ivr*-R	GGAGCCCGTGTAGAGCATGACGATC	Plant (endogenous)	invertase gene		95°C×5 min	
					94°C×30 s	
					61.5°C×45 s	
					72°C×45 s	
					72°C×10 min	
*phy*A2-F	AACACTCTCGATCCGGGCACCT	Plant (transgenic)	*phy*A2	678	94°C×5 min	Chen, *et al.*, 2008
					94°C×30 s	
					61.5°C×45 s	
					72°C×45 s	
					72°C×10 min	
*phy*A2-R	ACCAAGACACGGACCAAAGGC	Plant (transgenic)	*phy*A2		94°C×5 min	
					94°C×30 s	
					61.5°C×45 s	
					72°C×45 s	
					72°C×10 min	
*ov*-F	TGAAGATGGAGG AAAAATACAACC	Animal (Poultry)	ovalbumin gene	396	94°C×3 min	Jennings, *et al.*, 2003
					94°C×30 s	
					55°C×30 s	
					72°C×45 s	
					72°C×10 min	
*ov*-R	TGCAGCAGATAA CAT ACT TTT CAT.	Animal (Poultry)	ovalbumin gene		94°C×3 min	
					94°C×30 s	
					55°C×30 s	
					72°C×45 s	
					72°C×10 min	

1 PCR conditions for *ivr* F & R included 1 cycle at 95°C for 5 min, 40 cycles of 94°C for 30 s, down to 61.5°C for 45 s and back up to 72°C for 45 s and 1 cycle of 72°C for 10 min; *phy*A2-F & R; 1 cycle at 94°C for 5 min, 40 cycles of 94°C for 30 s, down to 61.5°C for 45 s and back up to 72°C for 45 s and 1 cycle of 72°C for 10 min; *ov*-F & R; 1 cycle at 94°C for 3 min, 35 cycles of 94°C for 30 s, down to 55°C for 30 s and back up to 72°C for 45 s and 1 cycle of 72°C for 10 min.

The PCR products were separated on a 1.5% agarose gels in 1×TBE (89 mM Tris, pH 8.4; 89 mM boric acid; 2 mM EDTA) and were visualized by ethidium bromide staining (Cowin Biotech, Beijing, China). The gel images were digitally captured using AlphaImager TM 2200 & 1220 Documentation & Analysis Systems (Alpha Innotech Corporation, San Leandro, CA).

### 8. Western blot analysis

Prior to protein extraction, all the samples were prepared by finely grinding in liquid nitrogen using clean, sterile pestles and mortars. Proteins were extracted from corns, diets and digesta using plant protein extraction reagent (Cowin Biotech, Beijing, China) and 5 µL PMSF (Sigma-Aldrich). The tissues were homogenized in 500 µL tissue protein extraction reagent (Cowin Biotech, Beijing, China) and 5 µL PMSF (Sigma-Aldrich). Supernatants were transferred into fresh tubes for SDS-PAGE and western blotting analysis. Protein concentration was determined by the BCA assay (Pierce Biotechnology, Rockford, IL, USA).

Protein samples were separated by 10% sodium dodecyl sulphate–polyacrylamide gel electrophoresis kit (SDS-PAGE gel kit; Cowin Biotech, Beijing, China) and then transferred to nitrocellulose membranes (Bio-Rad, USA). The blot was probed with a polyclonal antibody raised in rabbits against the yeast-expressed *phy*A2 protein [11]. Purification and characterization of the *phy*A2 protein followed the procedure as described elsewhere [11], [25]. The antibody was diluted by 2,000× for western blot analysis. The goat anti-rabbit IgG labeled with alkaline phosphatase (LI-COR Bioscience, Lincoln, NE, USA) were used as the second antibody.

### 9. Statistical analysis

All data were analyzed using the one-way analysis of ANOVA (SAS Institute, Inc., 2001). Differences between the two groups were analyzed according to *t*-tests. Effects were considered significant when *P*<0.05. Data are expressed as mean ± SD.

## Results

### 1. Effect of long-term feeding PTC on laying performance and egg quality

There were no adverse effects of feeding PTC on the laying performance and egg quality (*P*>0.05) of laying hens except on yolk color (Table 3). The yolk color was significantly decreased in the treatment fed with GM-maize diet (*P*<0.05) (Table 3).

**Table 3:** Performance and egg quality of laying hens fed diets containing control corn (CC) and phytase transgenic corn (PTC) for 16 weeks^1^.

Item	CC	PTC	*P*-value
Performance			
Egg weight (g/egg)	61.74±1.93	61.65±0.94	0.90
Egg production (%)	87.80±3.94	87.93±3.00	0.94
Egg mass (g of egg produced/d)	54.26±3.38	54.20±2.06	0.97
Feed intake (g/hen)	113.52±1.99	113.29±1.49	0.80
Feed efficiency (g of egg/g of feed)	2.10±0.13	2.09±0.06	0.90
Soft-shelled, cracked and broken eggs (%)	1.33±0.35	1.07±0.37	0.18
Mortality (%)	1.39±3.93	1.39±3.93	1.00
Egg quality			
Egg shape index	1.35±0.04	1.37±0.04	0.10
Shell thickness (mm)	0.33±0.02	0.33±0.02	0.35
Eggshell strength (kg/cm^2^)	2.82±0.65	2.79±0.52	0.82
Yolk colour	8.28±0.87^a^	7.85±0.66^b^	0.02
Albumen height (mm)	6.77±1.40	6.60±0.92	0.51
Haugh unit	80.57±8.64	80.01±5.10	0.73

1 Data are expressed as mean ± SD (*n* = 8).

a,b Means within a row with no common superscripts differ significantly (*P*<0.05).

### 2. Transgenic *phy*A2 gene detection in digesta, blood, tissues, and eggs of laying hens

**Figure 1:**
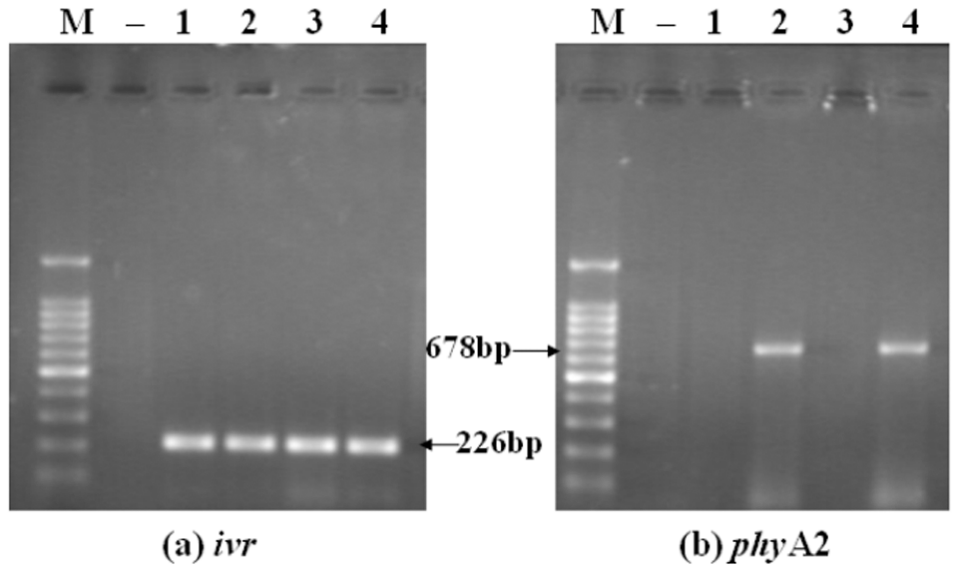
Fragmentation of DNA from control corn (CC) and phytase transgenic corn (PTC). (a) The endogenous gene (226 bp) of corn for *ivr* (left), (b) The exogenous gene (678 bp) of PTC for *phy*A2 (right). Arrows indicate the expected length of PCR products. M = marker; Lanes 1 = CC; lanes 2 = PTC; Lanes 3 = CC diet; lanes 4 = PTC diet; − = negative control (no DNA).

**Figure 2:**
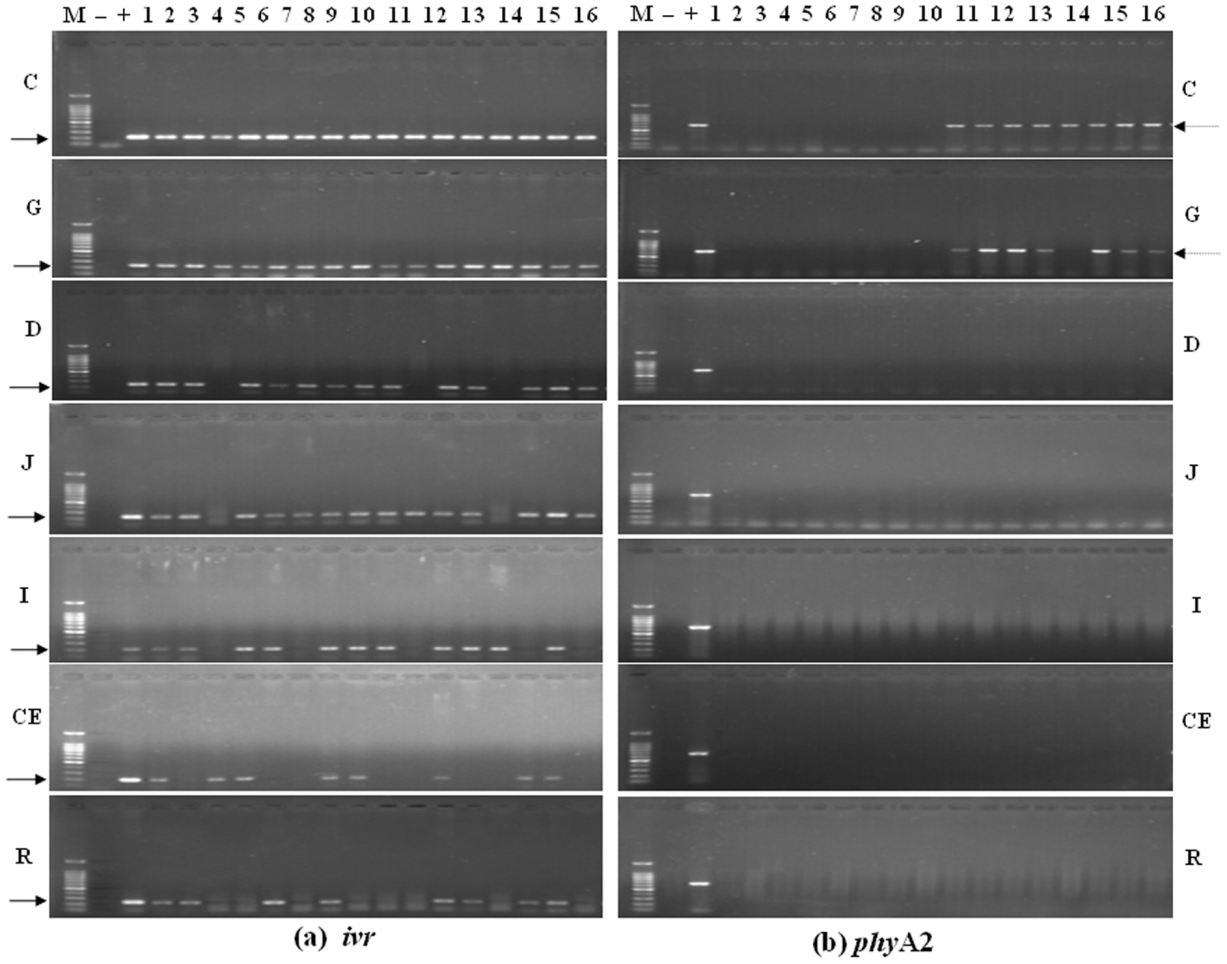
The PCR products from the gastrointestinal contents of laying hens fed diets containing control corn (CC) and phytase transgenic corn (PTC) for 16 weeks. (a) The endogenous gene (226 bp) of corn for *ivr* (left), (b) The exogenous gene (678 bp) of PTC for *phy*A2 (right). Arrows indicate the expected length of PCR products. M = marker; lanes 1 to 8 CC-fed laying hens (control); lanes 9 to 16 = PTC-fed laying hens; + = positive (GM corn), and − = negative control (no DNA). C = crop contents, G = gizzard contents, D = duodenal contents, J = jejunum contents, I = ileum contents, CE = cecal contents, and R = rectal contents.

PCR studies were used to find genes from corn, diets, digesta, blood, tissues, and eggs, either endogenous gene such as the corn specific invertase gene (*ivr*), specific poultry gene (*ov*), or transgenic fragments (*phy*A_2_). All the corn and diet samples were positive for *ivr* gene, while the transgenic *phy*A2 gene was only detected in PTC and PTC based diet (Figure 1). The *ivr* gene was also detected in all of the gastrointestinal sectors taken from both the CC and PTC-fed laying hens (Figure 2), while the *phy*A2 gene was only detected in upper GIT digest of laying hens fed with the PTC based diets (Table 4). Further down the GIT, the endogenous maize gene was detected in a relatively high frequency in along the length of the GIT in both the CC and PTC-fed laying hens (duodenum, 87.5% and 75%; jejunum, 87.5%; ileum, 75%; cecum, 62.5 and 50%; rectum, 62.5 and 50%, respectively) (Table 5). The *phy*A2 gene was not detected in duodenum digesta and the lower GIT of PTC-fed laying hens. The endogenous poultry gene (*ov*) was also detected in all digesta of laying hens fed CC or PTC based diet except in jejunum digesta non-GM maize-fed (87.5%) (Table 5).

**Table 4:** Detection of endogenous maize, poultry specific genes and transgenic *phy*A2 gene in digesta samples of laying hen fed diets containing control corn (CC) and phytase transgenic corn (PTC) for 16 weeks^1^.

Fragment amplified	Digesta^2^													
	Crop		gizzard		duodenum		jejunum		ileum		cecum		rectum	
	−	+	−	+	−	+	−	+	−	+	−	+	−	+
Endogenous														
*ivr* (corn)	8	8	8	8	7	6	7	7	6	6	5	4	5	4
*ov* (poultry)	8	8	8	8	8	8	7	8	8	8	8	8	8	8
Transgenic														
*phy*A2 (corn)	0	8	0	7	0	0	0	0	0	0	0	0	0	0

1 Number of samples that tested positive for the gene of interest out of 8 samples analyzed. One sample was tested per laying hen (n = 8 laying hens per treatment).

2 Treatments: “−” denotes CC-fed laying hens and “+” denotes PTC-fed laying hens.

C = crop contents, S = gizzard contents, D = duodenal contents, J = jejunum contents, I = ileum contents; CE = cecal contents, and R = rectal contents.

**Table 5:** Detection of the transgenic *phy*A2 gene in corn, diet, digesta, tissues, blood, and eggs of laying hens fed diets containing control corn (CC) and phytase transgenic corn (PTC) for 16 weeks^1^.

Item	Number of positive samples		Positive detection frequency in CC or PTC fed laying hens (%)^2^	
	CC	PTC	CC	PTC
Corn	0	8	0	100
Diet	0	8	0	100
Crop contents	0	8	0	100
Gizzard contents	0	7	0	87.5
Duodenal contents	0	0	0	0
Jejunum contents	0	0	0	0
Ileum contents	0	0	0	0
Cecal contents	0	0	0	0
Rectal contents	0	0	0	0
Heart	0	0	0	0
Liver	0	0	0	0
Spleen	0	0	0	0
Kidney	0	0	0	0
Breast muscle	0	0	0	0
Blood	0	0	0	0
Eggs	0	0	0	0

1 Number of samples that tested positive for the *phy*A2 gene out of 8 samples analyzed. One sample was tested per laying hen (n = 8 laying hens per treatment).

2 Percentage of samples positive for the *phy*A2 gene taken from PTC fed laying hens i.e. (number of positive samples/number of samples tested)×100.

**Figure 3:**
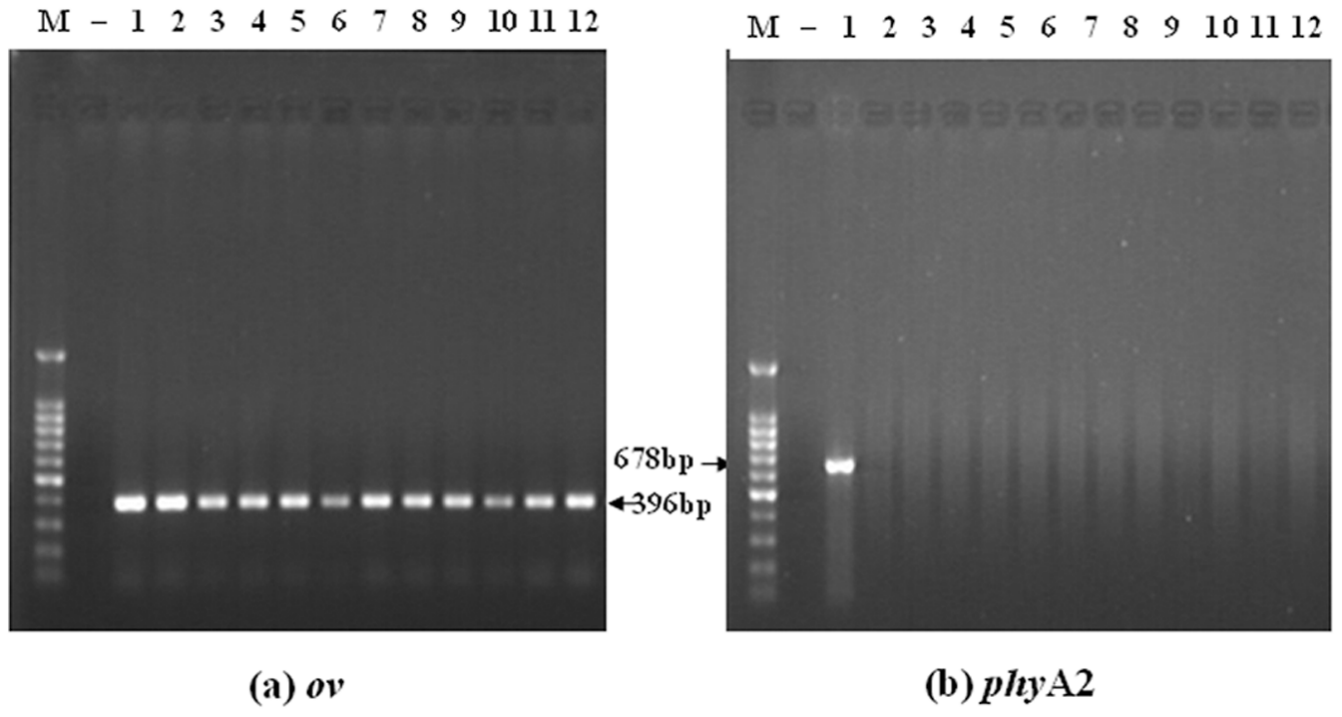
The PCR products from the tissues of laying hens fed diets containing control corn (CC) and phytase transgenic corn (PTC) for 16 weeks. (a) The endogenous gene (396 bp) of poultry for *ov* (left), (b) The exogenous gene (678 bp) of PTC for *phy*A2 (right). Arrows indicate the expected length of PCR products. M = marker; Lanes 1∼6 = blood, heart, liver, spleen, kidney, breast muscle of CC-fed laying hens (control); lanes 7∼12 = blood, heart, liver, spleen, kidney, breast muscle of PTC-fed laying hens; − = negative control (no DNA).

**Figure 4:**
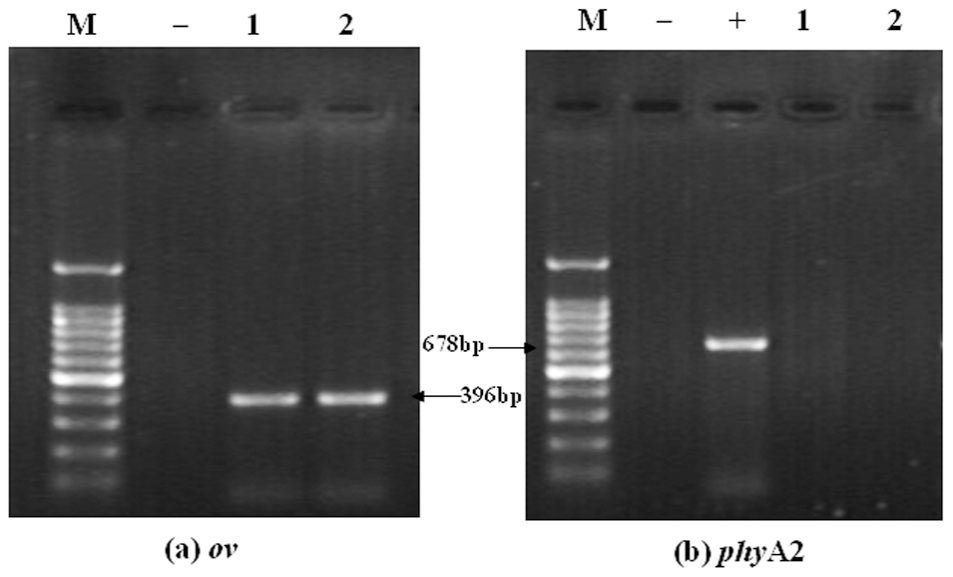
The PCR products from the egg contents of laying hens fed diets containing control corn (CC) and phytase transgenic corn (PTC) for 16 weeks. (a) The endogenous gene (396 bp) of poultry for *ov* (left), (b) The exogenous gene (678 bp) of PTC for *phy*A2 (right). Arrows indicate the expected length of PCR products. M = marker; lanes 1 = CC-fed laying hens (control); lanes 2 = PTC-fed laying hens; + = positive (GM corn), and − = negative control (no DNA).

No transgenic *phy*A2 gene fragments were detected in the blood, heart, liver, spleen, kidneys, breast muscle, and examined eggs (Table 5). However, as expected, all of the blood, tissue (Figure 3) and egg (Figure 4) samples were positive for the endogenous (*ov*) poultry gene in both the CC and PTC-fed group.

### 3. The *phy*A2 protein detection in digesta, blood, tissues, and eggs of laying hens

**Figure 5:**
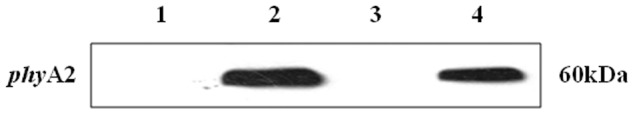
Western blot identification of *phy*A2 protein in corns and diets. Lane 1, control corn (CC); lane 2, phytase transgenic corn (PTC); lane 3, CC diet; lane 4, PTC diet.

**Figure 6:**
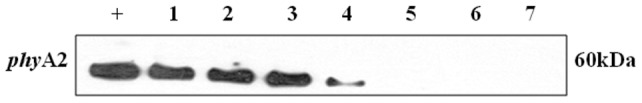
Western blot identification of *phy*A2 protein in gastrointestinal digesta of laying hens fed diet phytase transgenic corn for 16 weeks. “+” Positive control (GM corn); lane 1, crop contents; lane 2, gizzard contents; lane 3, duodenal contents; lane 4, jejunum contents; lane 5, ileum contents; lane 6, cecal contents; lane 7, rectal contents.

No *phy*A2 protein was detected in CC and CC diets, while the *phy*A2 protein was detected in all PTC and PTC diets (Figure 5). Likewise, no *phy*A2 protein was detected in all of the GIT digesta of laying hens fed CC based diet (Figure 6). The *phy*A2 protein was, however, detected in the 100% of crop samples and 87.5% of gizzard samples taken from PTC-fed laying hens (Table 6). Further down the GIT, the *phy*A2 protein was also detected in the lower gastrointestinal tract of PTC-fed laying hens, but only in the duodenal and jejunum samples (37.5% and 12.5%, respectively) and at a lower frequency than in the gizzard samples (Table 6).

**Table 6:** Western blot detection of the *phy*A2 protein in corn, diet, digesta, blood, tissues, and eggs of laying hens fed diets containing control corn (CC) and phytase transgenic corn (PTC) for 16 weeks^1^.

Item	Number of positive samples		Positive detection frequency in CC or PTC fed laying hens (%)^2^	
	CC	PTC	CC	PTC
Corn	0	8	0	100
Diet	0	8	0	100
Crop contents	0	8	0	100
Gizzard contents	0	7	0	87.5
Duodenal contents	0	3	0	37.5
Jejunum contents	0	1	0	12.5
Ileum contents	0	0	0	0
Cecal contents	0	0	0	0
Rectal contents	0	0	0	0
Blood	0	0	0	0
Heart	0	0	0	0
Liver	0	0	0	0
Spleen	0	0	0	0
Kidney	0	0	0	0
Breast muscle	0	0	0	0
Eggs	0	0	0	0

1 Number of samples that tested positive for the *phy*A2 protein out of 8 samples analyzed. One sample was tested per laying hen (n = 8 laying hens per treatment).

2 Percentage of samples positive for the *phy*A2 protein taken from PTC fed laying hens i.e. (number of positive samples/number of samples tested)×100.

**Figure 7:**
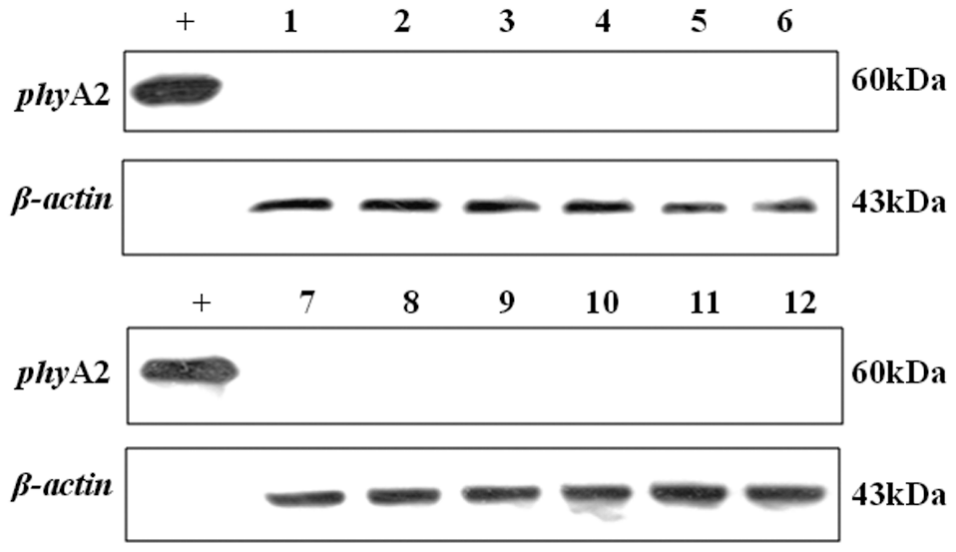
Western blot identification of *phy*A2 protein in tissues of laying hens fed diets containing phytase transgenic corn for 16 weeks. “+” Positive control (GM corn); non-transgenic group: lane 1, blood; lane 3, heart; lane 5, liver; lane 7, spleen; lane 9, kidney; lane 11, breast muscle. Transgenic group: lane 2, blood; lane 4, heart; lane 6, liver; lane 8, spleen; lane 10, kidney; lane 12, breast muscle.

**Figure 8:**
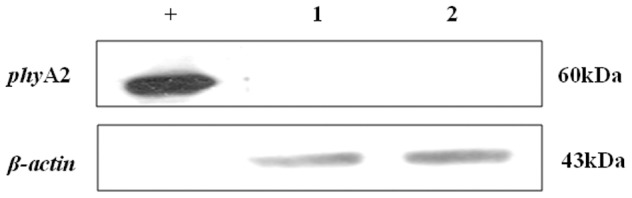
Western blot identification of *phy*A2 protein in eggs of laying hens fed diet containing phytase transgenic corn for 16 weeks. “+” Positive control (GM corn); lane 1, non-transgenic group; lane 2, transgenic group.

No *phy*A2 protein was detected in the blood, heart, liver, spleen, kidneys, breast muscle (Figure 7), and eggs (Figure 8) of laying hens fed either CC or PTC based diets (Table 6).

## Discussion

### 1. Effects of long-term feeding with PTC on laying performance and egg quality

The PTC was modified to improve the utilization of phytate-P derived from plant feedstuffs and decrease P excretion in feces [11]. Our previous study demonstrated that, the PTC used in the study has a higher phytase and fat contents, lower phytate contents than the CC [12]. No difference was observed in amino acid values between the PTC and CC [12]. The present results strongly suggest that there are no significant effects of the treatment in the measured growth performance parameters of feed intake, feed conversion efficiency, or mortality. This finding agrees with the results of previous studies that have confirmed laying hen performance is not affected by GM plants as compared to their conventional counterpart [26], [27]. Comparative studies [28], [29] with broilers fed GM maize and its isogenic non-GM varieties also showed that there were no adverse effects on the growth performance parameters. Likewise, no significant difference in growth performance parameters of pigs [30], steers [31], cows [32], fishes [33], or rats [34] receiving a non-GM diet or a GM plant-based diet has also been reported.

The egg quality also showed no statistically significant differences in egg shape index, shell thickness, eggshell strength, albumen height, and Haugh unit between laying hens consuming diets produced with PTC and CC. These results are consistent with laying hens feeding trials [27], [35], [36], which demonstrated similar egg quality between groups of laying hens fed diets with non-GM or GM maize. Although a decrease in yolk color was observed in PTC group compared with the control diet group, it may be explained by the higher levels of lutein in CC than in PTC (11.69 vs. 6.43 mg/kg). This is because yolk color is primarily contributed by the contents of lutein and carotene in diet [37].

In common with numerous researches, it indicates that PTC prepared from GM had no unintended effects on production traits and egg quality of laying hens as compared to CC.

### 2. Transgenic *phy*A2 gene detection in digesta, blood, tissues, and eggs of laying hens

#### 2.1 Digesta samples

DNA is the basic substance of all life and nearly all food or feed contains DNA. During the consumption of feed or food, DNA is sensitive to the digestive enzymes and gastric acid, and most of DNA is broken into subunits in digestive tract. Although all DNA, transgenic or not, is sensitive to the digestive processes, the DNA stability may be different in animals GIT [38], [39]. In this study, the degradation of *ivr* gene was detected to investigate whether the DNA degradability of a GM plant showed any difference compared with an unmodified plant. PCR data showed that *ivr* recovery, calculated as percentage of positives over all the samples, was recovered with high efficiency in all of the gastrointestinal sectors of non-GM and GM groups. No significant effect of two different diets (transgenic and non-transgenic) was observed on the *ivr* recovery throughout the GIT. However, the detection of the *phy*A2 genes in the digesta of PTC-fed laying hens decreased during passage through the GIT from 100% recovery in the crop and gizzard to undetectable in the small intestine and rectum. Similar findings have been reported by Chambers et al. [38], who demonstrated that transgenic gene was detected in the crop and gizzard but not in distal GIT of chickens fed GM maize. Therefore, the transgenic *phy*A2 genes appeared to be rapidly degraded as it passes through animals' GIT. These genetic materials were probably too degraded to allow amplification of such size of sequences in GIT of laying hens [15], [17]. Moreover, the *phy*A2 gene fragments could not be found probably due to their rare occurrence compared with *ivr* genes in the GIT.

Only strong positive signals for the *phy*A2 gene were detected in the PTC, PTC based diet and some digesta samples in GM group and no positive signals for control group. This indicates that cross-contamination between the two group could be excluded.

#### 2.2 Blood, tissues and eggs samples

Even though the *phy*A_2_ gene is rapidly degraded in GIT in this study, it seems possible that the DNA could be protected against degradation by certain dietary compounds in the upper GIT [40]. The possibility that DNA fragments were transported from bloodstream through GIT epithelia to host organism or eggs cannot be eliminated. In ileostomists small bowel, for example, DNA survives long enough to be capable of transforming a human GIT microflora [41]. Likewise, Nemeth *et al.* [42] and Sharma *et al.* [43] found that DNA fragments can cross the intestinal barrier and be detected in animal tissues and products.

In this study, we analyzed the blood and tissues such as heart, liver, spleen, kidneys and breast muscle of laying hens to demonstrate the absorption of gene fragments into the host organism. Moreover, DNA traceability was tested on genomic DNA from total eggs. In all bird blood, tissues and eggs to a PCR assays for the poultry specific ovalbumin (*ov*) gene was successfully amplified, assuring the suitability of the extraction and amplify ability of DNA. In contrast, no transgenic gene (*phy*A2) or maize specific gene (*ivr*) was detected in any of bird blood, tissues and eggs samples of the non-GM and GM group. This finding is consistent with numerous studies investigating the fate of transgenic DNA in animal blood or tissues [44], [45], [46], [47]. A rapid degradation throughout the GIT takes place [48], which might account for the absence of recombinant DNA in blood or tissues after feed intake.

Similar to the finding reported by Aeschbacher *et al.* [26], transgenic plant DNA was not detected in the eggs of laying hens. A possible explanation for this observation is that the eggs contain only a small amount of DNA [26]. Consequently, the low number of copies of gene may also hinder to trace a possible transfer of recombinant DNA into the eggs.

### 3. The *phy*A2 protein detection in digesta, blood, tissue, and eggs of laying hens

#### 3.1 Digesta samples

The *phy*A2 protein was detected in 100% of crop samples, 87.5% of gizzard samples, 37.5% duodenum samples and 12.5% jejunum samples taken from PTC-fed laying hens. Similar to the findings documented by Ash *et al.* [49], the detection of recombinant protein in the digesta of GM soybean-fed laying hens decreased during passage through the GIT from 100% recovery in the diet to undetectable in the feces. The possible explanation for the degradation of recombinant protein could be the enzymatic proteolysis and microbial in stomach and small intestine. However, Chowdhury *et al.* [44] reported that high levels of Cry1Ab protein were detected in rectal digesta of pigs, which was ascribed to incomplete degradation of recombinant protein in the GIT of pigs. This may reflect differences in the physiology of the GIT of avian and pigs. Birds have a gizzard where the surface area of the feed may be increased by grinding, which may further expose protein both to acid and to GIT proteolytic enzymes. In addition, susceptibility of the phytase protein to proteolytic cleavage and pH in the crop and gizzard [50], resulted in the drastic degradation of phytase protein in the crop and gizzard.

The discrepancy in detection frequencies between the transgenic gene and protein at similar sites along the GIT has also been reported by Walsh *et al.* [15], [17]. The authors observed that the chance of detection of transgenic genes is lower than the protein detection frequency further down the GIT (ileal, cecum, and colon). The use of primers with shorter expected fragment lengths increased transgenic gene detection frequency may account for the discrepancies observed in detection frequencies between the transgenic gene and protein in GIT [44], [51]. In the future, it is most likely that these transgenic genes or proteins can be detected at very low concentrations with improved methods.

#### 3.2 Blood, tissues and eggs samples

Similar to previous findings [15], [52], recombinant protein was not detected in the blood or any animal tissue samples. Moreover, our finding that no *phy*A2 protein was detected in the eggs is consistent with studies demonstrating that no transgenic CP4 EPSPS protein were found in the whole egg or albumin of laying hens receiving GM diets [48]. This is not surprising, considering that the transgenic genes were also undetectable in blood, tissues and eggs of laying hens. The genetic material was probably degraded by proteolytic enzymes in GIT, making them beyond the level of detection in the tissues and blood of laying hens. To date, no studies have reported the discovery of recombinant protein in blood, tissues, eggs or milk derived from animals fed GM-based diets.

In conclusion, the results obtained from long-term feeding with PTC to laying hens indicate that no adverse effects on laying performance and egg quality. Transgenic *phy*A2 gene and protein were detected in GIT digesta but can be rapidly degraded before excreted to the environment. There was no evidence of *phy*A2 gene or protein translocation to blood, tissues or, eggs of laying hens.
